# Sexual (Dis)satisfaction and Its Contributors Among People Living with HIV Infection in Sweden

**DOI:** 10.1007/s10508-017-1106-2

**Published:** 2018-02-13

**Authors:** Lena Nilsson Schönnesson, Galit Zeluf, Diego Garcia-Huidobro, Michael W. Ross, Lars E. Eriksson, Anna Mia Ekström

**Affiliations:** 1 Department of Public Health (Global Health/IHCAR), Karolinska Institutet, Widerströmska huset, Tomtebodavägen 18A, 171 77 Stockholm, Sweden; 2 Departamento de Medicina Familiar, Escuela de Medicina, Pontificia Universidad Católica de Chile, Santiago, Chile; 3 Program in Human Sexuality, Department of Family Medicine and Community Health, University of Minnesota Medical School, Minneapolis, MN USA; 4 Department of Learning, Informatics, Management and Ethics, Karolinska Institutet, Stockholm, Sweden; 5 School of Health Sciences, City, University of London, London, UK; 6 Department Infectious Diseases, Karolinska University Hospital, Stockholm, Sweden

**Keywords:** HIV, PLHIV, Sexual (dis)satisfaction, Sweden

## Abstract

Earlier research reports lower sexual satisfaction among people living with HIV (PLHIV) compared to HIV-negative persons. A number of psychosocial factors directly associated with sexual dissatisfaction have been identified. Little is known about sexual satisfaction and their contributors among PLHIV in Sweden. The aim of this study was to examine direct and indirect effects of variables within sociodemographic, clinical HIV-related, psychological, and sexual domains on sexual (dis)satisfaction among PLHIV in Sweden. Data for this study were derived from a nationally representative, anonymous survey among PLHIV conducted in 2014 (*n* = 1096). Statistical analysis included four steps: descriptive analyses, identification of variables associated with sexual (dis)satisfaction, identification of variables associated with those contributors of sexual (dis)satisfaction, and a path model integrating all these analyses. A total of 49% of participants reported being sexually dissatisfied, and no significant differences were observed when non-heterosexual men, heterosexual men, and women were compared. Among women, a negative change in sex life after HIV diagnosis and distress with orgasmic difficulties was directly associated with sexual dissatisfaction. For men, hopelessness, high HIV stigma, sexual inactivity in the last 6 months, and a negative change in sex life after HIV diagnosis were directly associated with sexual dissatisfaction. Path analyses showed in both men and women significant indirect associations between not being involved in an intimate relationship, lower self-reported CD4 cell counts, and perceiving obligation to disclose HIV status to sexual partners as a barrier to look for a long-term partner and sexual dissatisfaction. Our results show that despite good treatment outcomes, the HIV diagnosis has a negative bearing on sexual satisfaction. The need for gender-tailored interventions and clinical implications of these findings are discussed.

## Introduction

With advancements in antiretroviral therapy (ART), people living with HIV (PLHIV) in countries with general access to such treatment can live long and productive lives. Increased life expectancy of PLHIV and the transformation of HIV into a chronic condition call for the assessment of factors that are important for the quality of life in this group. The sexual life domain of PLHIV is one important dimension of quality of life characterized by various challenges (Skevington, Norweg, & Standage, 2010), which may have a bearing on PLHIVs sexual relationships and intimacy (Schönnesson & Ross, 1999; Siegel & Schrimshaw, 2003; Siegel, Schrimshaw, & Lekas, 2006) and sexual satisfaction.

Despite the fact that high adherence to ART leads to suppressed viral loads and minimal HIV transmission risk (World Health Organization, 2013), PLHIV may still fear both transmitting HIV to an intimate partner and being turned down sexually when disclosing their HIV status (Driskell, Salomon, Mayer, Capistrant, & Safren, 2008; Galletly & Dickson-Gomez, 2009). Another challenge is potential side-effects on sexual functioning due to ART (Asboe et al., 2007; Moreno-Pérez et al., 2010). Legal surveillance is a further potential challenge. Canadian qualitative studies (Kapiriri, Tharao, Muchenje, Masinde, & Ongoiba, 2016; Mykhalovskiy, 2011) have found that as a consequence of Canadian criminalization of HIV non-disclosure to sexual partners, some PLHIV withdraw from sexual activity due to fear of HIV transmission, anxiety, uncertainty, fear of abandonment, and loss of ability to engage in fulfilling sexual relationships.

In Sweden, HIV is a notifiable disease and subject to mandatory partner notification (The Public Health Agency of Sweden, 2015a, b). Obligation to disclose HIV status to sexual partners, regardless of condom use, is legalized in the Communicable Disease Act (see Folkhalsomyndigheten.se [The Public Health Agency of Sweden]). Such a legal obligation is thought to be counterproductive in that it puts the entire responsibility of practicing safer sex on PLHIV (Kaida et al., 2015). However, new evidence in 2013 regarding the minimal transmission risk of HIV in patients with undetectable viral load (< 20 copies/ml) (World Health Organization, 2013) has led to certain modifications in the implementation of the Swedish obligation to disclose HIV status to sexual partners. Individuals who fulfill the treatment requirements for viral suppression and who use a condom during vaginal or anal intercourse can be exempted from disclosing their HIV status (Swedish Institute for Infectious Disease Control, 2013). These exemptions are, however, regulated on an individualized basis by the patient’s physician and not all PLHIV in Sweden are aware of these potential modifications.

### Sexual Satisfaction Among PLHIV

Sexual satisfaction is an important element of sexual health and is associated with overall well-being. According to the World Health Organization’s definition, sexual health is “…not merely the absence of disease, dysfunction or infirmity. Sexual health requires a positive and respectful approach to sexuality and sexual relationships, as well as the possibility of having pleasurable and safe sexual experiences, free of coercion, discrimination and violence” (World Health Organization, 2016). Despite this holistic definition, sexual satisfaction is often discussed as synonymous to sexual functioning (Henderson, Lehavot, & Simoni, 2009). However, there is no consistent conceptual definition of sexual satisfaction, partially due to its subjective, socially constructed nature. One of the most accepted definitions is the one proposed by Lawrance and Byers (1995): “…an affective response arising from one’s subjective evaluation of the positive and negative dimensions associated with one’s sexual relationship.”

The assessment of sexual satisfaction among PLHIV varies across studies; in some studies, it is measured in terms of sexual dysfunctions as diagnosed in the International Classification of Diseases (ICD10) and DSM-IV-TR, in others by a Likert-scale question based on self-declared satisfaction with sex (Kaida et al., 2015), or by using scales which assess various dimensions of sexual life such as sexual sensations, sexual presence, sexual exchange, emotional connection, and sexual activity (Rodriguez-Diaz, Jovet-Toledo, Ortiz-Sanchez, Rodriguez-Santiago, & Vargas-Molina, 2015). del Mar Sánchez-Fuentes, Santos-Iglesias, and Sierra (2014) point out that used instruments are, with two exceptions (Lawrance & Byers, 1995; Stulhofer, Busko, & Brouillard, 2010), not based on theoretical conceptualizations of sexual satisfaction.

While PLHIV, as stated earlier, are confronted with sexual challenges, it has, not surprisingly, been documented that sexual satisfaction is lower in PLHIV than in HIV-negative persons (Wilson et al., 2010). The proportions of PLHIV reporting sexual satisfaction vary from 40% in Japan (Inoue, Yamazaki, Seki, Wakabayashi, & Kihara, 2004) to 48% in Great Britain, 61% in France (Rojas Castro, Le Gall, Andreo, & Spire, 2010), 64% in Canada (Kaida et al., 2015), and 68% in Romania (Lazar et al., 2014).

### Factors Associated with Sexual Dissatisfaction Among PLHIV

HIV-related research has identified a number of psychosocial factors negatively associated with sexual satisfaction. Not having a sex life is associated with greater sexual dissatisfaction (Beutel, Schumacher, Weidner, & Brahler, 2002; Lazar et al., 2014; Rojas Castro et al., 2010). In fact, a non-negligible proportion of PLHIV surveyed has reported an insignificant or non-existent sexual life and subsequently is sexually dissatisfied (Rojas Castro et al., 2010; Wilson et al., 2010). Non-active sexual life in PLHIV seems to be explained, at least partially, by HIV-related stigma and discrimination in the sexual setting (De Ryck, Van Laeken, Nostlinger, Platteau, & Colebunders, 2012; Kaida et al., 2015; Rojas Castro et al., 2010; Wilson et al., 2010). However, the association between stigma and sexual satisfaction is not fully understood (Bouhnik, Preau, Schiltz, Obadia, & Spire, 2008). Other psychosocial factors linked to sexual dissatisfaction include unemployment (Rojas Castro et al., 2010; Wilson et al., 2010), not having a steady relationship (Rodriguez-Diaz et al., 2015; Rojas Castro et al., 2010), low self-efficacy and social exclusion (Lazar et al., 2014), and feelings of loneliness (Rojas Castro et al., 2010; Wilson et al., 2010). Depression and anxiety were also found to be associated with sexual dissatisfaction in men living with HIV (De Ryck et al., 2012) and with sexual functioning among women living with HIV (Florence et al., 2004).

It has been suggested that improving sexual satisfaction in PLHIV may encourage preventive attitudes (Rojas Castro et al., 2010; Wilson et al., 2010) and to contribute to overall well-being (Anderson, 2013). It is therefore of public health importance to ensure that PLHIV have the opportunities to navigate a healthy sexual life. However, existing public health policies and programmes worldwide often fail to respond to the sexual (and reproductive health-related) rights, needs and aspirations of PLHIV (Gruskin, Ferguson, & O’Malley, 2007; Nöstlinger, Rojas Castro, Platteau, Dias, & Le Gall, 2014).

Little is known about sexual satisfaction and its contributors among PLHIV in Sweden. In addition, previous research has focused on direct effects of psychosocial factors, whereas, to our knowledge, no previous studies in Sweden, or internationally, have studied their potential indirect effects on sexual (dis)satisfaction.

### The Present Study

To address the importance of this topic and to expand the literature, this study aimed to examine direct and indirect effects of variables within sociodemographic, clinical HIV-related, psychological, and sexual domains on sexual (dis)satisfaction reported by PLHIV in Sweden.

Data for this study were derived from a large national, cross-sectional study “Living with HIV infection in Sweden” designed to examine self-reported quality of life and its correlates (The Public Health Agency of Sweden, 2015a, b). The study is the first of its kind in Sweden covering all subgroups of PLHIV across the country. Information about the study was posted on a website distributed by the Karolinska Institutet. Hard copies of the information were posted in waiting rooms at participating infectious disease outpatient clinics, and data were collected with an anonymous, self-reported questionnaire, distributed between December 2013 and August 2014.

## Method

### Participants

Participants were enrolled from 15 Swedish infectious disease outpatient clinics accounting for 75% of the HIV care in the country as well as from two needle and syringe exchange clinics in Stockholm in order to make sure a representative sample of injecting drug users was reached. Eligible participants were outpatients at least 18 years old and who had been diagnosed with HIV at least 6 months previously. The number of participants recruited at each participating clinic was pre-decided and proportional to the total number of patients living with HIV being followed at each clinic, corresponding to about 25% of all patients per unit. The aim to reach a representative sample of approximately 15% was accomplished; the 1096 included participants corresponded to 17% of all PLHIV in Sweden at the time of data collection. Overall, the sample represented all subpopulations living with HIV in Sweden, with regard to gender, age, HIV transmission route, and country of birth with the exception of a minor underrepresentation of persons reporting a heterosexual transmission route.

### Procedure

During the recruitment period, all consecutive PLHIV visiting their HIV unit were informed about the study and invited by a site coordinator to anonymously and voluntarily participate in the study by completing an anonymous questionnaire in their language of choice. Participants could complete the questionnaire in a confidential setting at their outpatient clinic, put the questionnaire in a sealed envelope, and drop it in a locked box (or complete it at home and mail it in a pre-stamped envelope to the research team at the Karolinska Institutet). The site coordinator collected the sealed envelopes and sent them to the research team at the Karolinska Institutet. No incentive was given. Participants were informed orally and by written information that a response to the anonymous questionnaire indicated that (s)he had consented to participate in the study.

Data were collected using an anonymous questionnaire prepared by the research team at the Karolinska Institutet in collaboration with the Public Health Agency, the main non-governmental organizations for PLHIV in Sweden, and the participating clinics. Representatives from the participating clinics and the non-governmental organizations gave feedback on the questionnaire, including 77 questions, before it was piloted during summer 2013 through the organizations. Completion of the questionnaire took between 20 and 40 min.

To reduce the risk of underrepresentation of migrant groups, the questionnaire was made available in 10 languages: Swedish, English, French, Spanish, Russian, Thai, Somali, Amharic, Arabic, and Tigrinya. The vast majority of the participants (82%) answered the questionnaire in Swedish and 8% in English. Our impression was that several participants actively dropped Somali, Tigrinya, Thai, Amharic, and Arabic in favor of Swedish or English, probably because of fear to be identified despite the fact the questionnaire was anonymous. Those who could not fill out the questionnaire in any of the languages or those being illiterate but still wanted to participate were offered to have the questions read to them by a telephone interpreter in a confidential setting at the respective collaborating infectious disease outpatient clinic. However, none of the participants chose this service.

### Measures

Based on prior research literature, variables derived from the questionnaire potentially associated with sexual satisfaction were divided into four domains, the sociodemographic, the clinical HIV-related, the psychological, and the sexual domain.

#### Dependent Variable

##### Sexual Satisfaction

Among all participants, regardless of sexual activity or inactivity in the past 6 months, sexual satisfaction was assessed using a six-point scale item from the Life Satisfaction Scale (Fugl-Meyer, Bränholm, & Fugl-Meyer, 1991): “With my sex life I am…..” (from “very dissatisfied” to “very satisfied”). As there is no gold standard to define sexual (dis)satisfaction, we chose an arbitrary cutoff at “rather dissatisfied.” The sexual satisfaction scale was thus dichotomized (satisfied/dissatisfied) for analyses.

##### Independent Variables

Independent variables of interest were grouped into sociodemographic, clinical HIV-related, psychological, and sexual domains.

##### Sociodemographic Domain

Sociodemographic characteristics included gender, age, relationship status (not in an intimate relationship/in an intimate relationship), level of education (< 1 year, 1–6 years, 7–9 years, 10–12 years, ≥ 13 years), employment status (working, non-working, retired/sick leave, studying), country of birth (born in Sweden/outside Sweden), self-reported sexual orientation (heterosexual, bisexual, homosexual/gay), and year of HIV diagnosis. With regard to “gender” the question was “Do you identify yourself as a. man, b. woman, c. other.” The variable “employment status” was dichotomized into “working or studying” and “non-working or non-studying” (also including retired/sick leave), “years of school” into < 12 years and ≥ 13 years, “self-reported sexual orientation” into “heterosexual” and “non-heterosexual,” and “year of HIV diagnosis” into < 1–10 years and 10–> 20 years.

##### Clinical HIV-Related Domain

Participants were asked to report their current CD4 cell count (< 100, 100–200, 200–350, 350–500, > 500 cells x 10^6^/l), whether they were taking ART at the moment (yes/no), and whether they experienced any physical and psychological side-effects, respectively, of their HIV treatment (yes/no). The CD4 cell count is a key measure of the status of the immune system. A normal CD4 count is from 500 to 1400 cells per cubic millimeter of blood. HIV attacks the immune system and lowers the CD4 count increasing the risk of serious illnesses, primarily opportunistic infections and cancers. CD4 cell counts were dichotomized into “low CD4 cell counts” (< 100–350) and “high CD4 cell counts” (350–> 500).

##### Psychological Domain

Two psychological constructs were used to measure psychological distress. Hopelessness is one of the chore characteristics of depression (Beck, 1963) reflecting negative expectations concerning oneself and one’s future. It was measured by the self-reported Beck Hopelessness Scale (Beck, Weissman, Lester, & Trexler, 1974). The timeframe was last seven days. It includes 9 positive and 11 negative true/false statements about the future (Cronbach’s alpha = 0.91 in the present study, mean = 5.44, median = 4.00, SD = 5.14). After reverse scoring of positively worded items, items were summed to give a total score ranging from 0 to 20, with a higher score reflecting increased hopelessness (score 0–3 “absence of hopelessness”; score 4–8 “mild hopelessness”; score 9–12 “moderate hopelessness”; and score > 12 “severe hopelessness”). Hopelessness was then dichotomized into “absent” and “present” (mild, moderate, severe).

Post-traumatic stress disorder (PTSD) symptoms related to HIV diagnosis were measured using the Impact of Event Scale (Horowitz, Wilner, & Alvarez, 1979). The scale measures current (within the last week) symptoms of intrusive thoughts (e.g., Did you think about HIV when you did not want to?) and avoidant thoughts and behaviors (e.g., I tried not to talk about HIV) of PTSD. It contains 15 questions, rated on a four-point scale “not at all,” “rarely,” “sometimes,” and “often” (Cronbach’s alpha = 0.94 in the present study, mean = 22.66, median = 19.00, SD = 18.85). Items were summed to provide a total score from 0 to 75 points, with higher scores indicating more severe HIV-related PTSD symptoms (score 0–8 “subclinical”; score 9–25 “mild,” score 26–43 “moderate,” and score > 44 “severe”). HIV-related PTSD symptoms were dichotomized into “subclinical” and “present” (mild, moderate, severe).

HIV-related stigma was measured by selected items from a short-form of the Swedish version (Lindberg, Wettergren, Wiklander, Svedhem-Johansson, & Eriksson, 2014; Reinius et al., 2017) of the HIV Stigma Scale (Berger, Ferrans, & Lashley, 2001). The present study focused on two dimensions of HIV stigma, negative self-image/internalized stigma (3 items: I feel guilty about having HIV; The attitudes of others about HIV make me think less of myself; and Having HIV makes me feel inferior to others) and one aspect of anticipated HIV stigma, Concerns about public attitudes toward PLHIV (3 items: People with HIV are treated like lepers; Most people think that someone with HIV is dirty; and Most people I know feel uncomfortable being around someone who has HIV). Each item was rated on a four-point scale ranging from “strongly disagree” (1) to “strongly agree” (4) (Reinius et al., 2017). Items of Negative self-image/internalized stigma were summed to a total score ranging from 3 to 12 (Cronbach’s alpha = 0.80 in the present study, mean = 6.45, median = 6.00, SD = 2.76), higher scores reflecting increased experienced HIV-related stigma. Items of concerns about public attitudes toward PLHIV were summed in the same way (Cronbach’s alpha = 0.82, mean = 7.71, median = 8.00, SD = 2.58). Scores between 3 and 7 in the respective scale were considered “low HIV-related stigma” and scores between 8 and 12 as “high HIV-related stigma.”

##### Sexual Domain

The sexual domain refers here to four aspects of sexuality. The first aspect related to sexual activity, importance of sex, sexual desire, and sexual pleasure. The second aspect illustrated whether the HIV diagnosis had changed participants’ sex life. The third aspect highlighted potential consequences of the obligation to disclose one’s HIV status to sexual partners on participants’ sex life. The fourth aspect examined level of distress with sexual difficulties (Lewin, Fugl-Meyer, Helmius, Lalos, & Månsson, 1998). See Table 1 for a description of each of the variables and their response categories.

**Table 1:** Description of sexual domain variables

Questionnaire question	Response categories
Have you had sex with anyone in the past 6 months	Yes/no
How important is sex to you?	Very important
	Fairly important
	Not very important
	Not important at all
	Dichotomized into:
	Very/fairly important
	Not very important/not important at all
Everyone’s sexual desire varies from time to time. How often have you felt sexual desire in the past 6 months?	Never
	Rarely
	Sometimes
	Often
How often have you experienced pleasure in the past 6 month when masturbating or having sex with a partner?	Never
	Rarely
	Sometimes
	Often
	Always
Has being HIV-positive changed your sex life?	Positive change
	No change
	Negative change
Has somebody turned you down sexually in the past 6 months because you have HIV?	Yes/no
Does the obligation to inform people that you have HIV affect your sex life (you can check more than one)?	I have fewer partners
	I don’t have sex anymore
	I usually have sex with other HIV-positive people
	I can’t enjoy sex as much anymore
	It helps me to tell potential partners right away that I have HIV
	It does not affect my sex life at all
	An information obligation index was computed divided into two categories:
	Low impact
	High impact
Does the obligation to inform sexual partner that you have HIV prevent you from finding a long-term partner?	Definitely
	To some extent
	Not at all
	Dichotomized into:
	Definitely/to some extent
	Not at all
Are you ever worried that somebody you had sex with will report you to Institute for Communicable Disease control?	All the time
	Often
	Once in a while
	Never
Are you ever worried that somebody you had sex with will report you to the police?	All the time
	Often
	Once in a while
	Never
It is difficult for women to have orgasms sometimes. Have you had that experience in the past 6 months?	Never/almost never
	Rarely
	Sometimes
	Often
	Always
	Dichotomized into:
	Never/rarely
	Sometimes/often/always
It is difficult for men to have erection/hard-on sometimes. Have you had that experience in the past 6 months?	Never/almost never
	Rarely
	Sometimes
	Often
	Always
	Dichotomized into:
	Never/rarely
	Sometimes/often/always
It is difficult for men to ejaculate sometimes. Have you had that experience in the past 6 months?	Never/almost never
	Rarely
	Sometimes
	Often
	Always
	Dichotomized into:
	Never/rarely
	Sometimes/often/always
Has this [orgasmic difficulties, erectile difficulties, and ejaculatory difficulties, respectively] been a problem for you in your sexual life in the past 6 months?	No problem at all
	A minor problem
	A major problem
	Dichotomized into:
	No distress
	Minor or major distress

### Statistical Analyses

In total, 1096 eligible individuals responded to the anonymous questionnaire of whom 762 were men, 320 were women, four participants reported “other” gender identity, and ten participants did not report their gender. Fourteen participants (4 “other” and 10 missing) were dropped from further analysis, leaving a total of 1082 participants. As the correlates of sexuality are gender dependent (Siegel & Schrimshaw, 2003), we stratified all our analyses by gender.

The statistical analyses were performed with MPlus version 7.4 and included four steps: descriptive analyses, identification of variables associated with sexual (dis)satisfaction, identification of variables associated with those contributors of sexual (dis)satisfaction, and a path model integrating all of these analyses. All analyses of male participants were adjusted by self-reported sexual orientation.

First, descriptive statistics were calculated to present demographic and clinical HIV-related characteristics of the female and male participants using mean with standard deviation for continuous variables and frequency (%) for categorical variables. Second, to identify variables associated with sexual (dis)satisfaction, sociodemographic, clinical HIV-related, psychological, and sexual contributors were studied one at a time using binary logistic regression. Unadjusted odds ratio (OR) and its 95% confidence intervals (95% CI) were calculated. Then, all variables that had a statistically significant association with sexual (dis)satisfaction in the binary logistic regression models were included in a multiple logistic regression to examine which variables explained sexual (dis)satisfaction while holding other potential predictors constant. ORs and 95% CIs were calculated.

The third step aimed to identify variables of domains that could indirectly contribute to sexual (dis)satisfaction by being associated with the variables that were directly and statistically related to the dependent variable of interest. First, binary linear and logistic regressions (for continuous and categorical dependent variables, respectively) were conducted between the sociodemographic, clinical HIV-related, psychological, and sexual variables and those variables that were identified as statistically associated with sexual (dis)satisfaction. Unadjusted ORs and 95% CIs were calculated for variables predicting categorical outcomes. Statistically significant variables were then included in multiple linear or logistic regression models, and adjusted ORs and 95% CIs were calculated for categorical independent variables.

The final analytical step evaluated the fit of the direct and indirect contributors of sexual (dis)satisfaction. Separate path analyses were developed for female and male participants using only the statistically significant variables from previous analyses using maximum likelihood estimators. With maximum likelihood and categorical variables, means, variances, and covariances are not sufficient statistics for model estimation, so chi-square and related fit statistics are not available (Muthén, 1993). Full models were compared to null models using the likelihood difference test, where -2 times the loglikelihood difference is distributed as chi-square (Browne & Cudeck, 1993), and differences in Akaike Information Criteria (AIC) and Bayesian Information Criteria (BIC).

#### Missing data

Complete data were available for 222 male participants (29.1%) and 52 female participants (16.3%). Data on sexual (dis)satisfaction were available for 985 (91.0%) participants. To manage different degrees of missing data, we used multiple imputation followed by maximum likelihood estimation (Muthén & Muthén, 2015). Imputation for variables with missing values was conducted using Bayesian analyses (Rubin, 1987). Ten imputed datasets were used in the estimation of all analyses using maximum likelihood estimation. Maximum likelihood parameter estimates for each analysis were averaged over the set of 10 analyses, and standard errors were computed using the average of the standard errors of the analyses and the between analyses parameter estimation. Multiple imputation and maximum likelihood estimations are the best methods to manage missing data when performing data analysis (Graham, 2012).

The following variables were excluded from analyses due to high skewness (< − 1 or > 1): “self-reported ART,” “self-reported psychological side-effects of ART,” “turned down sexually in the past 6 months,” “worries to be reported by a sex partner to the authorities due to not adhering to the obligation to disclose one’s HIV status to sexual partners,” and the “obligation to HIV disclosure index.”

## Results

### Demographic and Health Characteristics

Table 2 reports descriptive data on sociodemographic and clinical HIV-related characteristics. Overall, most participants were men (70%) aged on average 50 years, and were in an intimate relationship (55%). Men were mostly non-heterosexual (64%), while women were mostly heterosexual (94%). The vast majority (99%) of the self-identified homosexual/gay men/MSM who were involved in an intimate relationship reported a male partner. Among the bisexual men, over one half (59%) reported a female partner and 41% a male intimate partner. Among the eight non-heterosexual women (self-identified as bisexual) who reported having an intimate partner, five had a male and three a female partner. The majority of the women were born outside of Sweden. HIV transmission through sexual contact with men was the most common route among men as well as among women.

**Table 2:** Sociodemographic and clinical HIV-related characteristics

Variable	Men (*n* = 762) (%)/mean (SD)	Women (*n* = 320) (%)/mean (SD)
*Sociodemographic domain*		
Age (years)	49.81 (11.61)	42.46 (10.50)
Current relationship status		
Not in an intimate relationship	342 (45%)	118 (38%)
In an intimate relationship	414 (55%)	196 (62%)
Years of school		
< 12 years	379 (51%)	186 (59%)
> 13 years	365 (49%)	128 (41%)
Employment		
Non-working or non-studying	159 (24%)	85 (28%)
Working or studying	508 (76%)	214 (72%)
Country of birth		
Sweden	506 (68%)	87 (28%)
Outside Sweden	244 (32%)	228 (72%)
Self-reported sexual orientation		
Heterosexual	258 (36%)	219 (94%)
Non-heterosexual	449 (64%)	14 (6%)
HIV transmission route		
Sexual contact with men	58%	61%
Sexual contact with women	25%	–
Needles/syringes	10%	13%
Blood products	5%	10%
From mother	–	3%
Year of HIV diagnosis		
< 1–10 years	372 (50%)	178 (56%)
10–> 20 years	375 (50%)	136 (44%)
*Clinical HIV-related domain*		
Self-reported current CD4 cells		
< 100–350	152 (31%)	58 (32%)
> 350–> 500	335 (69%)	124 (68%)
Self-reported ART		
No	25 (3%)	17 (5%)
Yes	727 (97%)	300 (95%)
Self-reported physical side-effects of ART		
No	497 (71%)	184 (65%)
Yes	203 (29%)	99 (35%)

*ART* antiretroviral therapy

### Sexual Satisfaction

Almost half of the participants (49%) (men 50% and women 44%) reported being sexually dissatisfied. No significant differences were observed when non-heterosexual men, heterosexual men and women were compared.

### Binary Logistic Regression Analyses Predicting Sexual (Dis)satisfaction

Regarding women, the binary logistic regression analysis did not show any significant associations between variables within the sociodemographic, clinical HIV-related, and the psychological domains and sexual (dis)satisfaction (Table 3). In relation to female participants’ sexual life, experiencing distress with orgasmic difficulties, and reporting that HIV had changed their sex life negatively were significantly associated with sexual dissatisfaction.

**Table 3:** Binary and multiple logistic regressions predicting sexual (dis)satisfaction, male (*n* = 762) and female (*n* = 320) participants

Variable	Binary logistic regression		Multiple logistic regression	
	Male participants^a^ OR (95% CI)	Female participants OR (95% CI)	Male participants^a^ OR (95% CI)	Female participants OR (95% CI)
*Sociodemographic domain*				
Age	1.00 (0.99, 1.02)	0.97 (0.92–1.03)		
Relationship status				
Not in an intimate relationship	0.35*** (0.25, 0.47)	0.63 (0.24–1.64)	0.59 (0.30, 1.16)	
In an intimate relationship	1	1	1	
Years of school				
< 12 years	0.87 (0.65, 1.17)	1.13 (0.50–2.55)		
≥ 13 years	1	1		
Employment				
Non-working or non-studying	0.56** (0.38, 0.84)	1.18 (0.69–2.03)	1.11 (0.46, 2.68)	
Working or studying	1	1	1	
Country of birth				
Sweden	1	1		
Outside Sweden	0.94 (0.69, 1.28)	0.68 (0.25–1.87)		
Year of HIV diagnosis				
≤ 10 years	1.10 (0.82, 1.47)	1.24 (0.52–2.97)		
> 10 years	1	1		
*Clinical HIV-related domain*				
Self-reported physical side-effects of ART				
No	1	1	1	
Yes	2.02*** (1.44, 2.83)	2.04 (0.79–5.27)	0.95 (0.48, 1.88)	
Self-reported current CD4 cells				
< 350	0.71 (0.49, 1.04)	1.12 (0.33–3.88)		
350–500	1	1		
*Psychological domain*				
Hopelessness				
Absent	1	1	1	
Present	0.21*** (0.15, 0.30)	0.84 (0.36–1.94)	0.35*** (0.22, 0.48)	
HIV-related PTSD symptoms				
Subclinical	1	1		
Present	0.80 (0.57, 1.12)	1.39 (0.59–3.32)		
HIV stigma: concerns about public attitudes toward PLHIV				
Low stigma	1	1	1	
High stigma	0.46*** (0.34, 0.61)	0.44 (0.17–1.15)	0.49*** (0.18, 0.67)	
HIV stigma: negative self-image				
Low stigma	1	1		
High stigma	0.86 (0.63, 1.16)	1.06 (0.45–2.47)		
*Sexual domain*				
Sex in the past 6 months				
Sexual inactivity	0.17*** (0.12, 0.25)	0.33 (0.10–1.06)	0.12*** (0.05, 0.29)	
Sexual activity	1	1	1	
Importance of sex				
Very or fairly important	1	1	1	
Not very important or not important at all	1.52* (1.07, 2.17)	0.94 (0.40–3.00)	0.61 (0.23, 1.43)	
Sexual desire in the past 6 months	1.78*** (1.46, 2.17)	1.11 (0.54–2.27)	1.05 (0.68, 1.63)	
Sexual pleasure in the past 6 months	1.96*** (1.67, 2.30)	1.43 (0.80–2.57)	2.04*** (1.34, 3.10)	
Erectile difficulties				
Never or rarely	1	–	1	
Sometimes, often, or always	0.69* (0.50, 0.93)		0.76 (0.40, 1.44)	
Distress with erectile difficulties		–		
No distress	1		1	
Minor or major distress	0.69* (0.48, 0.99)		1.23 (0.43, 2.54)	
Orgasmic difficulties	–		–	
Never or rarely		1		
Sometimes, often, or always		1.93 (0.60–6.22)		
Distress with orgasmic difficulties	–		–	
No distress		1		1
Minor or major distress		0.22* (0.06–0.87)		0.47* (0.23, 0.97)
Ejaculatory difficulties		–		
Never or rarely	1		1	
Sometimes, often, or always	0.65* (0.46, 0.92)		1.17 (0.73, 1.86)	
Distress with ejaculatory difficulties				
No distress	1	–	1	
Minor or major distress	0.52*** (0.36, 0.76)		0.63 (0.33, 1.57)	
HIV changed sex life				
Positive change	2.49*** (1.55, 4.02)	1.91 (0.41–8.90)	0.42 (0.12, 1.42)	1.48 (0.48, 4.61)
No change	1	1	1	1
Negative change	0.16*** (0.11, 0.23)	0.12** (0.03–0.48)	0.06*** (0.02, 0.21)	0.07*** (0.03, 0.18)
Obligation to disclose HIV status an obstacle to look for a long-term partner				
Definitely or to a certain extent	0.35*** (0.25, 0.50)	0.73 (0.24–1.72)	2.05 (0.97, 3.30)	
Not at all	1	1	1	

*OR* odds ratio, *CI* confidence interval, *ART* antiretroviral therapy, *PLHIV* people living with HIV, *PTSD symptoms* post-traumatic stress disorder symptoms

* *p* < .05; ** *p* < .01; *** *p* < .001

^a^Adjusted by sexual orientation

For men, not being in a relationship and/or non-working or non-studying had a negative effect on sexual satisfaction. As to the clinical HIV-related domain, self-reported physical side-effects of ART were significantly associated with sexual satisfaction. With regard to psychological domain variables, hopelessness and high HIV stigma related to concerns about public attitudes toward PLHIV were significantly correlated with sexual dissatisfaction. The binary analysis showed a negative significant association of the following variables within the sexual domain with sexual satisfaction: sexual inactivity in the last 6 months, erectile and ejaculatory difficulties and their related distress in the past 6 months, negative HIV-related changes in sex life, and perceiving the obligation to disclose one’s HIV status to sexual partners as an obstacle to look for a long-term partner. Sex perceived as not important, high frequency of sexual desire and sexual pleasure, and positive HIV-related changes in sex life correlated with sexual satisfaction.

### Multiple Logistic Regression Analysis Predicting Sexual (Dis)satisfaction

Regarding women, the statistically significant variables in the binary logistic regression analysis remained statistically significant in the multiple logistic regression analysis; experiencing minor or major distress with orgasmic difficulties and HIV had changed their sex life negatively increased the likelihood of rating one’s sex life as unsatisfactory (Table 3).

Concerning men, variables from the psychological and sexual domains that remained significantly associated with sexual dissatisfaction in the multiple logistic regression analysis were hopelessness, HIV stigma related to concerns about public attitudes toward PLHIV, sexual inactivity in the last 6 months, and negative HIV-related changes in sex life. Sexual pleasure remained linked to sexual satisfaction.

### Multiple Regressions Models Predicting Sexual (Dis)satisfaction Mediators

For female participants, statistically significant variables associated with distress with orgasmic difficulties and with negative HIV-related changes in sex life, respectively, in the binary regression analysis were included in separate multiple regression models (Table 4). The results of the models showed that variables from the psychological and sexual domains for distress with orgasmic difficulties and variables from sociodemographic, clinical HIV-related, and sexual domains for negative HIV-related changes in sex life remained significant.

**Table 4:** Multiple regression models predicting sexual (dis)satisfaction mediators, male (*n* = 762) and female participants (*n* = 320)

Variable	Male participants^a^					Female participants	
	Hopelessness	High HIV stigma: public attitudes toward PLHIV	Sexual inactivity in last 6 months	Sexual pleasure in the past 6 months	Negative HIV-related changes in sex life	Distress with orgasmic difficulties	Negative HIV-related changes in sex life
	OR (95% CI)	OR (95% CI)	OR (95% CI)	B (95% CI)	OR (95% CI)	OR (95% CI)	OR (95% CI)
*Sociodemographic domain*							
Age	1.00 (0.99, 1.02)	0.99 (0.98, 1.01)	1.02** (1.00, 1.04)	0.00 (− 0.01, 0.01)	1.00 (0.98, 1.02)		
Relationship status							
Not in an intimate relationship	1.34 (91, 1.97)		4.28*** (2.82, 6.51)	− 0.32*** (− 0.45, − 0.18)	1.78* (1.13, 2.82)		2.57* (1.20, 5.48)
In an intimate relationship	1		1	0	1		1
Years of school							
< 12 years	1.33 (0.94, 1.87)						
≥ 13 years	1						
Employment							
Non-working or non-studying	0.97 (0.69, 1.37)		0.60** (0.44, 0.83)	− 0.03 (− 0.11, 0.04)	0.99 (0.67, 1.47)		
Working or studying	1		1	0	1		
Country of birth							
Sweden				0	1		1
Outside Sweden				− 0.21 (− 0.37, − 0.05)	0.43*** (0.27, 0.70)		0.25*** (0.11, 0.54)
Year of HIV diagnosis							
≤ 10 years		1.35 (0.97, 1.88)	0.67 (0.44, 1.02)		0.94 (0.63, 1.42)		
> 10 years		1	1		1		
*Clinical HIV-related domain*							
Self-reported physical side-effects of ART							
No	1	1	1		1		
Yes	0.69 (0.47, 1.01)	0.61** (0.43, 0.87)	0.61** (0.39, 0.95)		0.62 (0.38, 1.00)		
Self-reported current CD4 cells							
< 350			1.75** (1.10, 2.80)		2.60*** (1.45, 4.67)		0.26*** (0.11, 0.59)
350–500			1		1		1
*Psychological domain*							
Hopelessness							
Absent						1	
Present						2.52* (1.21, 5.22)	
HIV-related PTSD symptoms							
Subclinical			1		1	1	
Present			1.15 (0.74, 1.79)		1.46 (0.94, 2.26)	0.40* (0.17, 0.90)	
HIV stigma: concerns about public attitudes toward PLHIV							
Low stigma							
High stigma							
HIV stigma: negative self-image							
Low stigma						1	
High stigma						1.83 (0.88, 3.79)	
*Sexual domain*							
Sex in the past 6 months							
Sexual inactivity						0.22** (0.07, 0.68)	3.13* (1.13, 8.67)
Sexual activity						1	1
Sexual pleasure in the past 6 months						0.71* (0.52, 0.96)	0.70* (0.52, 0.94)
Sexual desire in the past 6 months	0.86 (0.70, 1.06)		0.54*** (0.41, 0.71)	0.76*** (0.67, 0.86)	0.70** (0.52, 0.92)		
Importance of sex							
Very or fairly important		1	1	0			
Not very important or not important at all		1.90*** (1.33, 2.72)	0.22*** (0.22, 0.57)	0.35*** (0.16, 0.53)			
Erectile difficulties							
Never or rarely	1			0	1		
Sometimes, often, or always	0.72 (0.41, 1.29)			− 0.16 (− 0.40, 0.09)	0.94 (0.48, 1.84)		
Distress with erectile difficulties							
Never or rarely	1		1	0	1		
Sometimes, often, or always	1.40 (0.76, 2.58)		0.42*** (0.25, 0.69)	0.15 (− 0.12, 0.42)	1.45 (0.70, 2.98)		
Ejaculatory difficulties							
Never or rarely	1			0	1		
Sometimes, often, or always	1.48 (0.89, 2.45)			− 0.10 (− 0.31, 0.11)	1.00 (0.54, 1.85)		
Orgasm difficulties							
Never or rarely						1	
Sometimes, often, or always						6.01*** (2.80, 12.91)	
Distress with ejaculatory difficulties							
Never or rarely	1			0	1		
Sometimes, often, or always	1.65 (0.93, 2.93)			− 0.39** (− 0.64, − 0.13)	1.39 (0.67, 2.92)		
Obligation to disclose HIV status an obstacle to look for a long-term partner							
Definitely or to a certain extent	2.42*** (1.53, 3.82)	2.00*** (1.41, 2.85)	1.54 (0.95, 2.50)		4.14*** (2.68, 6.39)		1.92 (0.99, 3.72)
Not at all	1	1	1		1		1

*OR* odds ratio, *CI* confidence interval, *ART* antiretroviral therapy, *PLHIV* people living with HIV, *PTSD symptoms* post-traumatic stress disorder symptoms

* *p* < .05; ** *p* < .01; *** *p* < .001

^a^Adjusted by sexual orientation

The male binary analysis showed that variables within the sociodemographic and the sexual domains were correlated with all five mediators (hopelessness, HIV stigma related to concerns about public attitudes toward PLHIV, sexual inactivity in the last 6 months, negative HIV-related changes in sex life, and sexual pleasure). Clinical HIV-related domain variables correlated with all mediators except sexual pleasure. Variables within the psychological domain associated with sexual inactivity and negative HIV-related changes in sex life. After including them in separate multiple regression models, all domains except the psychological one remained significant in the multiple regressions.

### Path Analyses

#### Female Participants

**Fig. 1:**
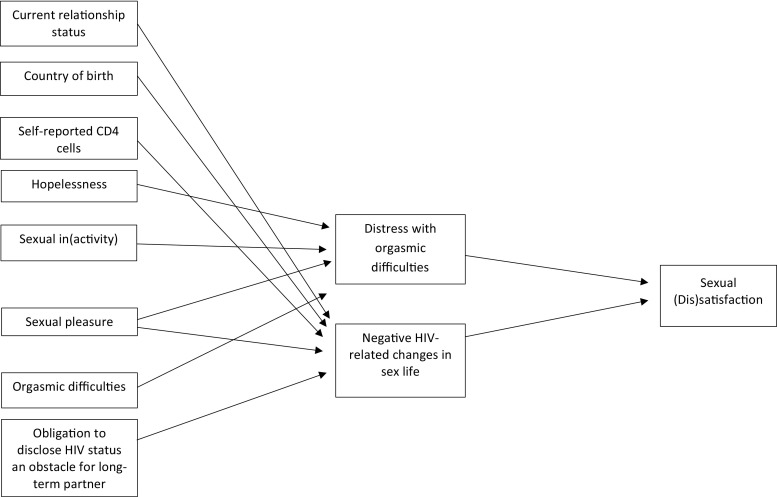
Path diagram, female participants (*n* = 320)

The model fit of the female path analysis showed a loglikelihood = − 2097.9, AIC = 4255.8, and BIC = 4368.86. This model had significantly better fit than an empty model (loglikelihood = − 2237.30; AIC = 4494.7; BIC = 4532.3). Figure 1 presents the model, and Table 5 shows path coefficients for different predictors. All variables remained significant in the full model highlighting indirect effects over sexual (dis)satisfaction. Table 5 presents the ORs or regression coefficients (95% CI) for the different paths.

**Table 5:** Path analysis for male (*n* = 762) and female (*n* = 320) participants

Male participants			Female participants		
Independent Variable	Dependent Variable	OR (95% CI)	Independent variable	Dependent variable	OR (95% CI)
Hopelessness	Sexual dissatisfaction	0.30*** (0.18, 0.50)	Distress with orgasmic difficulties	Sexual dissatisfaction	0.41* (0.19, 0.88)
High HIV stigma: public attitudes toward PLHIV	Sexual dissatisfaction	0.58* (0.36, 0.94)	Negative HIV-related changes in sex life	Sexual dissatisfaction	0.05*** (0.02, 0.14)
Sexual inactivity in last 6 months	Sexual dissatisfaction	0.15*** (0.08, 0.28)	Hopelessness	Distress with orgasmic difficulties	3.23** (1.55, 6.72)
Sexual pleasure	Sexual dissatisfaction	1.39* (1.07, 1.81)	Sexual inactivity in last 6 months	Distress with orgasmic difficulties	0.21** (0.08, 0.56)
Negative HIV-related changes in sex life	Sexual dissatisfaction	0.23*** (0.12, 0.42)	Low frequency of sexual pleasure	Distress with orgasmic difficulties	0.67** (0.49, 0.92)
Obligation to disclose HIV status an obstacle for long-term partner	Hopelessness	2.39*** (1.60, 3.57)	Orgasmic difficulties	Distress with orgasmic difficulties	5.69*** (2.76, 11.71)
Self-reported physical side-effects of ART	High HIV stigma: public attitudes toward PLHIV	0.61* (0.40, 0.93)	Not in an intimate relationship	Negative HIV-related changes in sex life	2.56* (1.12, 5.85)
Sex not very important/not important at all	High HIV stigma: public attitudes toward PLHIV	1.88** (1.21, 2.93)	Born outside Sweden	Negative HIV-related changes in sex life	0.30** (0.14, 0.65)
Obligation to disclose HIV status an obstacle for long-term partner	High HIV stigma: public attitudes toward PLHIV	2.05*** (1.37, 3.06)	Low self-reported CD4 cell count < 350	Negative HIV-related changes in sex life	0.27** (0.11, 0.68)
Not in an intimate relationship	Sexual inactivity in last 6 months	4.82**((2.96, 7.85)	Sexual inactivity in last 6 months	Negative HIV-related changes in sex life	3.26** (1.32, 8.06)
Self-reported physical side-effects of ART	Sexual inactivity in last 6 months	0.55* (0.33, 0.93)	Sexual pleasure	Negative HIV-related changes in sex life	0.70* (0.50, 0.96)
Sex not very important/not important at all	Sexual inactivity in the last 6 months	0.31*** (0.18, 0.54)	Obligation to disclose HIV status an obstacle to look for long-term partner	Negative HIV-related changes in sex life	2.31* (1.16, 4.62)
Sexual desire	Sexual activity in last 6 months	0.53*** (0.37, 0.74)			
Not in an intimate relationship	Low frequency of sexual pleasure	− 0.28** (− 0.45, − 0.11)			
Sex is very/fairly important	High frequency of sexual pleasure	0.37** (0.14, 0.59)			
High frequency of sexual desire	High frequency of sexual pleasure	0.78*** (0.65, 0.92)			
Distress with ejaculatory difficulties	Low frequency of sexual pleasure	− 0.37*** (− 0.57, − 0.16)			
Not in an intimate relationship	Negative HIV-related changes in sex life	1.96* (1.15, 3.37)			
Low self-reported CD4 cell count < 350	Negative HIV-related changes in sex life	2.12** (1.28, 3.52)			
Low frequency of sexual desire	Negative HIV-related changes in sex life	0.63* (0.45, 0.90)			
Obligation to disclose HIV status an obstacle for long-term partner	Negative HIV-related changes in sex life	4.48*** (2.69, 7.44)			

*OR* odds ratio, *CI* confidence interval, *ART* antiretroviral therapy, *PLHIV* people living with HIV

* *p* < .05; ** *p* < .01; *** *p* < .001

^a^Adjusted by sexual orientation

#### Male Participants

**Fig. 2:**
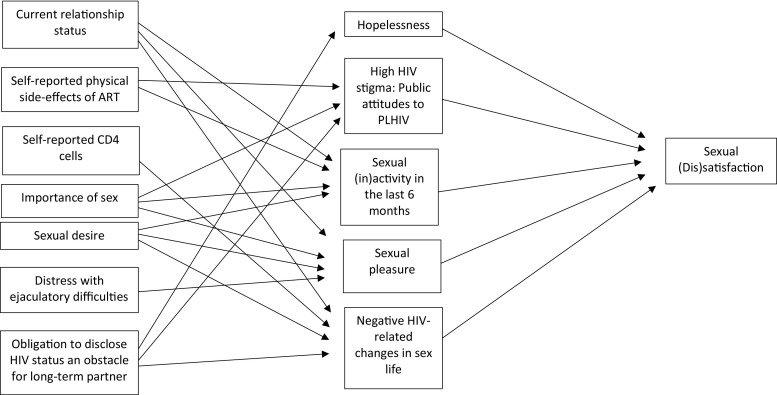
Path diagram, male participants (*n* = 762)

The model fit of the male path analysis showed a loglikelihood = −1940.6, AIC = 3949.5, and BIC = 4091.5. This model also had a significantly better fit than the empty model (loglikelihood = − 2312.7, AIC = 4651.4, and BIC = 4705.7). As shown in Fig. 2, the male participants have a complex web of indirect pathways to sexual (dis)satisfaction through hopelessness, high HIV stigma/concerns about public attitudes toward PLHIV, sexual inactivity in the last 6 months, sexual pleasure, and negative HIV-related changes in sex life. Table 5 presents the ORs or regression coefficients (95% CI) for the different paths.

## Discussion

This study, based on data from a national representative sample, is the first to examine satisfaction with sexual life and its direct and indirect correlates in PLHIV in Sweden and, as far as we are aware, elsewhere. Self-reported sexual dissatisfaction was found to be high as reported by other studies (Inoue et al., 2004; Rojas Castro et al., 2010; Weatherburn et al., 2009; Wilson et al., 2010). Sexual dissatisfaction is a response when sexual aspirations/goals exceed the achievement (Fugl-Meyer, Lodnert, Branholm, & Fugl-Meyer, 1997). No significant differences were observed when non-heterosexual, and heterosexual men and women were compared.

A larger percentage of PLHIV than in the general Swedish population declared being sexually dissatisfied. The “Sex in Sweden” study (Lewin et al., 1998), which had a random population sample using the same instrument as we did, reported that those rating their sex life as “very dissatisfied/dissatisfied” was 10%, whereas the percentage in our sample based on the same cut point “very dissatisfied/dissatisfied” was 33%. For our study, those at the “very dissatisfied/dissatisfied/rather dissatisfied” self-classifications constituted 49% of the sample. Swedish clinical data from a health questionnaire filled out by 926 patients living with HIV visiting their infectious disease outpatient clinics in 2012 found that 41% were sexually dissatisfied, using the same instrument as we did.

Our findings indicated that variables within sociodemographic, clinical HIV-related, psychological, and sexual domains contributed directly and indirectly to sexual dissatisfaction and were gender dependent. Before discussing the results, we want to highlight certain limitations of this study.

### Limitations

First, as our data are of cross-sectional character they preclude determination of causality but also how and in what way sexual (dis)satisfaction and its contributors may change over time. Second, self-reporting data may include the well-known social desirability, but it may have decreased as the questionnaire was anonymous. Third, the assessment of sexual satisfaction was only based on one item. As this study did not include HIV-negative women and men, it limits a broader understanding of the impact of HIV versus non-HIV factors on sexual satisfaction. Fourth, one reason for the complexity of the male, in contrast to the female, path diagram may be that as female participants were fewer, there was a lower power to discover associations in that group. Fifth, as the analyses included multiple comparisons, statistical significance may have been achieved just by chance indicating caution about the presented results.

Despite these limitations, data collected in this study confirmed results from prior research (De Ryck et al., 2012; Kaida et al., 2015; Siegel et al., 2006) that HIV infection is a burden to PLHIV in relation to their sexual life. In the following, we will summarize variables within each domain of particular interest.

### Sociodemographic Domain

#### Relationship Status

Not being involved in an intimate relationship was for women and men associated with the experience that their sex life had changed in a negative way and had indirectly a negative effect on sexual satisfaction. We do not know, based on our data, whether being single was of an intentional or circumstantial character, but irrespective of which one, our result supports other studies (Rodriguez-Diaz et al., 2015; Rojas Castro et al., 2010) albeit in an indirect way. One possible explanation of the finding is that there was a yearning for an intimate relationship, but HIV-related concerns such as disclosure put up barriers for fulfilling this wish and consequently PLHIV were sexually unsatisfied. In fact, emerging research acknowledges loneliness as a central aspect of life among PLHIV (Fekete, Williams, & Skinta, 2017; Siemon, Blenkhorn, Wilkins, O’Brien, & Solomon, 2013; Storholm et al., 2013; Vincent et al., 2016) and that loneliness is associated with HIV-related stigma (Fekete et al., 2017; Siemon et al., 2013; Storholm et al., 2013; Vincent et al., 2016). However, it cannot be excluded that non-HIV-related issues or circumstances may have played a role in not having an intimate relationship, and causal relationships could be postulated in either direction.

### Clinical HIV-Related Domain

In contrast to other studies (Kaida et al., 2015; Lambert, Keegan, & Petrak, 2005; Rojas Castro et al., 2010), our results indicated that for both women and men, lower CD4 cell counts were an indirect barrier through negative HIV-related changes in one’s sex life to a fulfilling sex life. It is quite likely that a more or less impaired immune system due to one’s HIV infection, one’s health is impaired, which in turn may circumscribe sexual encounters.

### Psychological Domain

HIV stigma did not play a role for sexual dissatisfaction in the female path diagram, but did so for male participants supporting earlier studies (Rojas Castro et al., 2010; Wilson et al., 2010). If the individual anticipates negative attitudes from others because of his HIV in combination with experiencing the Swedish obligation to disclose one’s HIV status to sexual partners as an obstacle to look for a long-term partner, it might stop him from getting involved in sexual encounters and subsequently be sexually dissatisfied.

### Sexual Domain

#### HIV Changed Sexual Life in a Negative Way

In concordance with other Scandinavian studies (Backer Grønningsæter, Mandal, Nuland, & Haug, 2009; Carstensen & Dahl, 2007), women and men experiencing that their sex life had changed in a negative way after the HIV diagnosis were sexually dissatisfied. After HIV diagnosis, the individual’s sex life alters and becomes surrounded by restrictions. One indirect barrier of a satisfying sex life was perceiving the obligation to disclose one’s HIV status to sexual partners as an obstacle to look for a long-term partner. Clinical experiences suggest that some PLHIV are so afraid to disclose their HIV status that they do not dare to reveal themselves to potential partners. To disclose one’s HIV status more or less always involves worries to be rejected or abandoned by sexual partners and is perceived as a potential source of frustration and stress. To be rejected as a sexual person is a hard blow for one’s self-image and self-esteem and may lead to feelings of being no-one (Schönnesson & Ross, 1999). We would argue that disclosure stress is reinforced by the obligation to disclose one’s HIV status, which in turn was associated with experiencing that one’s sex life had changed negatively after being diagnosed with HIV.

Our findings also showed that women being born outside Sweden were more likely than women born in Sweden to report that HIV had changed their sex life in a negative way. Many women with HIV, who live in Sweden but were not born here, express that they keep their HIV status as a secret due to worries of being stigmatized, discriminated against, and excluded from their communities if their HIV status would become “official.” To look for a sexual partner is thus of even greater concerns given the obligation to disclose one’s HIV status. Within such a context, it is understandable that non-Swedish-born women living with HIV reported that their sex life had changed in a negative way.

#### Sexual Inactivity in the Last 6 Months

Studies (Bernier et al., 2016; Bogart et al., 2006; Kaida et al., 2015; Wilson et al., 2010) have shown that sexual inactivity is common among PLHIV, in particular among women ranging from 18 to 49% (Bernier et al., 2016; Bogart et al., 2006; Kaida et al., 2015; Wilson et al., 2010). The prevalence of sexual inactivity among women in our study was 43% and among men 35% (data not shown). As shown elsewhere (Rojas Castro et al., 2010; Wilson et al., 2010), a potentially more or less obvious finding was that sexual inactivity in the last 6 months among men had a direct and for women an indirect negative effect on sexual satisfaction. The question here is whether withdrawal from or avoidance of sexual encounters could appear as a protective shield, i.e., as a coping strategy, not to be rejected and hurt. Nonetheless, it still had a negative effect on sexual satisfaction.

#### Sexual Difficulties and Related Distress

In concordance with sexological research (Stephenson & Meston, 2010; Velten & Margraf, 2017), sexual distress had a negative effect on sexual satisfaction. It is of note that sexual desire was neither a direct nor an indirect contributor to sexual satisfaction among women, although other studies indicate low sexual desire among women living with HIV (Carlsson-Lalloo, Rusner, Mellgren, & Berg, 2016).

#### Obligation to Disclose HIV Status to Sexual Partners

Perceiving the Swedish legal obligation to disclose one’s HIV status as a barrier to look for a long-term partner played a significant role for both women and men, albeit indirectly, in experiencing an unsatisfactory sex life. Despite recent relief in the implementation of the obligation to disclose HIV status to sexual partners, HIV disclosure is still perceived as problematic by PLHIV, particularly when looking for a steady relationship. The current exemption to disclose HIV status when fulfilling the clinical criteria of viral suppression and using a condom is by many PLHIV perceived as helpful when engaging in temporary/casual sex. However, anecdotal reports from clinicians working with PLHIV as well as members of non-governmental HIV organizations reveal that when a casual sexual relationship develops into a steady one, PLHIV want to be open about their HIV status but are afraid to be rejected due to non-disclosure of HIV at first encounter. As a consequence, some PLHIV may decide to refrain from a steady relationship.

We would suggest that legal and social boundaries, such as the obligation to disclose HIV status to sexual partners, may be perceived as an intrusion in the individual’s life or as an external control as they are imposed upon the individual by HIV. For men, the HIV disclosure obligation was also associated with HIV stigma related to concerns about public attitudes toward PLHIV.

### Conclusion

Sweden is the first country to achieve the joint United Nations Programme on 90-90-90 continuum of HIV care targets (Gisslén et al., 2017). Despite good treatment outcome (95% of PLHIV in Sweden who are on treatment have undetectable viral load), our results undoubtedly show that PLHIV are confronted with many challenges related to their sex life. As Kaida points out “good treatment outcomes alone aren’t enough to’normalize sex and intimacy” (Tng, 2016). Our findings indicate that to get a better understanding of the complexity of contributors to sexual dissatisfaction we need to pay attention to direct as well as indirect factors. Moreover, the study clearly shows the need for gender-based interventions regarding sexuality among PLHIV. Some contributors were clearly HIV-related, whereas for others it was not that clear. It could not be ruled out that for example not having a steady partner or feelings of hopelessness were unrelated to HIV.

HIV-related stigma has been emphasized to be a critical obstacle for initiating sexual relationship and may also inhibit sexual activity (Bernier et al., 2016; Platteau et al., 2015; Rojas Castro et al., 2010; Wilson et al., 2010). In our study, only men reported high HIV stigma. It is of note that negative self-image did not, but concerns about public attitudes toward PLHIV did correlate with sexual dissatisfaction. The result indicates that fear of rejection or being discarded because of one’s HIV status is far more prominent than HIV-related negative self-image within a sexual context. Our data clearly show the need for public health and sociostructural interventions/strategies to destigmatize HIV.

The obligation to disclose one’s HIV status to sexual partners was perceived by the majority of study participants as a source of frustration (data not shown). Such a structural factor is an additional form of stigmatization and compromises sexual satisfaction among PLHIV in Sweden.

These data also have clinical implications. Now that HIV is considered a chronic disease with a long-life expectancy, management of the sexual consequences of HIV has implications for both mental health, quality of life, and sexual well-being of women and men living with HIV. However, it is not uncommon there is a reluctance among HIV health care staff to address sexual issues. But also, PLHIV may by various reasons be hesitant to initiate such a discussion. For example, our data showed that 25% of those who scored sexual dissatisfaction wanted to talk about their sexual matters but had not brought it up with their doctor or nurse (data not shown).

The prevalence of sexual dissatisfaction is high in both men and women living with HIV. It underscores the importance of a positive response instead of silence in clinical and social work encounters. Staff should initiate a dialogue and offer counseling related to sexual satisfaction. Clinicians need to be knowledgeable about the complexity of contributors to sexual dissatisfaction to attempt, if possible, to minimize their impact. Not all are related to sexuality, but factors such as HIV treatment, impaired health, legal issues, stigma, psychological distress, and the HIV diagnosis in itself also play a role. Psychological/psychiatric as well as sexological referrals should be offered to the client when deemed appropriate. Inquiring about sexual satisfaction of a client sends both the message that sexuality is a concern of the clinician and that s/he has a positive outlook to sexuality issues. While some of the determinants of sexual dissatisfaction are beyond the control of the clinician, other responses such as raising the issue, demonstrating concern, and sex-positive interaction may reduce the client’s sense of hopelessness and feelings of sexual isolation. The ultimate goal should be to maximize an individual’s possibility to recognize herself/himself as a sexual human being who has the right to sexual well-being.

## References

[CR1] Anderson RM (2013). Positive sexuality and its impact on overall well-being. Bundesgesundheitsblatt-Gesundheitsforschung-Gesundheitsschutz.

[CR2] Asboe D, Catalan J, Mandalia S, Dedes N, Florence E, Schrooten W, Nöstlinger C, Colebunders R (2007). Sexual dysfunction in HIV-positive men is multi-factorial: A study of prevalence and associated factors. AIDS Care.

[CR3] Backer Grønningsæter, A., Mandal, R., Nuland, B. R., & Haug, H. (2009). *Living with HIV in Norway—2009* (No. Fafo-report 2009:43). Retrieved from http://www.fafo.no/media/com_netsukii/20103.pdf.

[CR4] Beck AT (1963). Thinking and depression. I. Idiosyncratic content and cognitive distortions. Archives of General Psychiatry.

[CR5] Beck AT, Weissman A, Lester D, Trexler L (1974). The measurement of pessimism: The Hopelessness scale. Journal of Consulting and Clinical Psychology.

[CR6] Berger BE, Ferrans CE, Lashley FR (2001). Measuring stigma in people with HIV: Psychometric assessment of the HIV Stigma scale. Research in Nursing & Health.

[CR7] Bernier A, Lefèvre M, Henry E, Verdes L, Acosta ME, Benmoussa A, Mukumbi H, Cissé M, Otis J, Préau M (2016). HIV seropositivity and sexuality: Cessation of sexual relations among men and women living with HIV in five countries. AIDS Care.

[CR8] Beutel ME, Schumacher J, Weidner W, Brahler E (2002). Sexual activity, sexual and partnership satisfaction in ageing men—Results from a German representative community study. Andrologia.

[CR9] Bogart LM, Collins RL, Kanouse DE, Cunningham W, Beckman R, Golinelli D, Bird CE (2006). Patterns and correlates of deliberate abstinence among men and women with HIV/AIDS. American Journal of Public Health.

[CR10] Bouhnik A-D, Preau M, Schiltz M-A, Obadia Y, Spire B (2008). Sexual difficulties in people living with HIV in France–Results from a large representative sample of outpatients attending French hospitals (ANRS-EN12-VESPA). AIDS and Behavior.

[CR11] Browne MW, Cudeck R, Bollen KA, Long JS (1993). Alternative ways of assessing model fit. Testing structural equation models.

[CR12] Carlsson-Lalloo E, Rusner M, Mellgren A, Berg M (2016). Sexuality and reproduction in HIV-positive women: A meta-synthesis. AIDS Patient Care and STDs.

[CR13] Carstensen, M., & Dahl, A. (2007). *HIV och levekår*—*en undersøgelse af hivsmittedes levekår og livskvalitet i Danmark: HIV*-*Danmark 2007.* Retrieved from http://www.hiv-danmark.dk/fileadmin/template/html/levekaarsfiler/pdf/Levekaar.pdf.

[CR14] De Ryck I, Van Laeken D, Nostlinger C, Platteau T, Colebunders R (2012). Sexual satisfaction among men living with HIV in Europe. AIDS and Behavior.

[CR15] del Mar Sánchez-Fuentes M, Santos-Iglesias P, Sierra JC (2014). A systematic review of sexual satisfaction. International Journal of Clinical and Health Psychology.

[CR16] Driskell JR, Salomon E, Mayer K, Capistrant B, Safren S (2008). Barriers and facilitators of HIV disclosure: Perspectives from HIV-infected men who have sex with men. Journal of HIV/AIDS & Social Services.

[CR17] Fekete EM, Williams SL, Skinta MD (2017). Internalized HIV-stigma, loneliness, depressive symptoms and sleep quality in people living with HIV. Psychology & Health.

[CR18] Florence E, Schrooten W, Dreezen C, Gordillo V, Schönnesson LN, Asboe D, Koitz G, Colebunders R (2004). Prevalence and factors associated with sexual dysfunction among HIV-positive women in Europe. AIDS Care.

[CR19] Fugl-Meyer AR, Bränholm I-B, Fugl-Meyer KS (1991). Happiness and domain-specific life satisfaction in adult northern Swedes. Clinical Rehabilitation.

[CR20] Fugl-Meyer AR, Lodnert G, Branholm IB, Fugl-Meyer KS (1997). On life satisfaction in male erectile dysfunction. International Journal of Impotence Research.

[CR21] Galletly CL, Dickson-Gomez J (2009). HIV sero-positive status disclosure to prospective sex partners and criminal laws that require it: Perspectives of persons living with HIV. International Journal of STD and AIDS.

[CR22] Gisslén M, Svedhem V, Lindborg L, Flamholc L, Norrgren H, Wendahl S, Sönnerborg A (2017). Sweden, the first country to achieve the joint United Nations Programme on HIV/AIDS (UNAIDS)/World Health Organization (WHO) 90–90-90 continuum of HIV care targets. HIV Medicine.

[CR23] Graham JW (2012). Missing data: Analysis and design.

[CR24] Gruskin S, Ferguson L, O’Malley J (2007). Ensuring sexual and reproductive health for people living with HIV: An overview of key human rights, policy and health systems issues. Reproductive Health Matters.

[CR25] Henderson AW, Lehavot K, Simoni JM (2009). Ecological models of sexual satisfaction among lesbian/bisexual and heterosexual women. Archives of Sexual Behavior.

[CR26] Horowitz M, Wilner N, Alvarez W (1979). Impact of Event scale: A measure of subjective stress. Psychosomatic Medicine.

[CR27] Inoue Y, Yamazaki Y, Seki Y, Wakabayashi C, Kihara M (2004). Sexual activities and social relationships of people with HIV in Japan. AIDS Care.

[CR28] Kaida A, Carter A, de Pokomandy A, Patterson S, Proulx-Boucher K, Nohpal A, Loutfy M (2015). Sexual inactivity and sexual satisfaction among women living with HIV in Canada in the context of growing social, legal and public health surveillance. Journal of the International AIDS Society.

[CR29] Kapiriri L, Tharao W, Muchenje M, Masinde KI, Ongoiba F (2016). ‘… They should understand why…’The knowledge, attitudes and impact of the HIV criminalisation law on a sample of HIV + women living in Ontario. Global Public Health.

[CR201] Lambert, S., Keegan, A., & Petrak, J. (2005). Sex and relationships for HIV positive women since HAART: A quantitative study. *Sexually Transmitted Infections,**81*, 333–337. 10.1136/sti.2004.013516 PMC1745005 16061542

[CR31] Lawrance K-A, Byers ES (1995). Sexual satisfaction in long-term heterosexual relationships: The Interpersonal Exchange Model of Sexual Satisfaction. Personal Relationships.

[CR32] Lazar F, Verdes L, Henry E, Fugon L, Bernier A, Otis J, Preau M (2014). Satisfaction with sexual life in people living with HIV in Romania, together with associated individual and social factors. AIDS Care.

[CR33] Lewin, B., Fugl-Meyer, K., Helmius, G., Lalos, A., & Månsson, S.-A. (1998). *Sex i Sverige; Om sexuallivet i Sverige* 1996. Folkhälsoinstitutet.

[CR34] Lindberg MH, Wettergren L, Wiklander M, Svedhem-Johansson V, Eriksson LE (2014). Psychometric evaluation of the HIV stigma scale in a Swedish context. PLoS ONE.

[CR35] Moreno-Pérez O, Escoín C, Serna-Candel C, Pico A, Alfayate R, Merino E, Reus S, Boix V, Sanchez-Paya J, Portilla J (2010). Risk factors for sexual and erectile dysfunction in HIV-infected men: The role of protease inhibitors. AIDS.

[CR36] Muthén B, Bollen KA, Long JS (1993). Goodness of fit with categorical and other non-normal variables. Testing structural equation models.

[CR37] Muthén LK, Muthén BO (2015). Mplus user’s guide.

[CR38] Mykhalovskiy E (2011). The problem of “significant risk”: Exploring the public health impact of criminalizing HIV non-disclosure. Social Science and Medicine.

[CR200] Nöstlinger, C., Rojas Castro, D., Platteau, T., Dias, S., & Le Gall, J. (2014). HIV-related discrimination in European health care settings. *AIDS Patient Care and STDS,**28*, 155–161. 10.1089/apc.2013.0247 PMC3948597 24568694

[CR39] Platteau T, Nöstlinger C, Schrooten W, Kenyon C, van Lankveld JJDM, Colebunders R, The Eurosupport Study Group (2015). Sexual inactivity among men who have sex with men living with HIV in Europe. International Journal of Sexual Health.

[CR40] Reinius M, Wettergren L, Wiklander M, Svedhem V, Ekström A-M, Eriksson LE (2017). Development of a 12-item short version of the HIV Stigma scale. Health and Quality of Life Outcomes.

[CR41] Rodriguez-Diaz CE, Jovet-Toledo GG, Ortiz-Sanchez EJ, Rodriguez-Santiago EI, Vargas-Molina RL (2015). Sexual health and socioeconomic-related factors among HIV-positive men who have sex with men in Puerto Rico. Archives of Sexual Behavior.

[CR42] Rojas Castro D, Le Gall JM, Andreo C, Spire B (2010). Stigma, discrimination, and sexual (dis)satisfaction among people living with HIV: Results from the “AIDES et toi” survey. AIDS Care.

[CR43] Rubin DB (1987). Multiple imputation for nonresponse in surveys.

[CR44] Schönnesson LN, Ross MW (1999). Coping with HIV infection: Psychological and existential responses in gay men.

[CR45] Siegel K, Schrimshaw EW (2003). Reasons for the adoption of celibacy among older men and women living with HIV/AIDS. Journal of Sex Research.

[CR46] Siegel K, Schrimshaw EW, Lekas H-M (2006). Diminished sexual activity, interest, and feelings of attractiveness among HIV-infected women in two eras of the AIDS epidemic. Archives of Sexual Behavior.

[CR47] Siemon JS, Blenkhorn L, Wilkins S, O’Brien KK, Solomon PE (2013). A grounded theory of social participation among older women living with HIV. Canadian Journal of Occupational Therapy.

[CR48] Skevington SM, Norweg S, Standage M (2010). Predicting quality of life for people living with HIV: International evidence from seven cultures. AIDS Care.

[CR30] Stephenson KR, Meston CM (2010). Differentiating components of sexual well-being in women: Are sexual satisfaction and sexual distress independent constructs?. Journal of Sexual Medicine.

[CR49] Storholm ED, Halkitis PN, Kupprat SA, Hampton MC, Palamar JJ, Brennan-Ing M, Karpiak S (2013). HIV-related stigma as a mediator of the relation between multiple-minority status and mental health burden in an aging HIV-positive population. Journal of HIV/AIDS & Social Services.

[CR50] Stulhofer A, Busko V, Brouillard P (2010). Development and bicultural validation of the new Sexual Satisfaction scale. Journal of Sex Research.

[CR51] Swedish Institute for Infectious Disease Control (2013). Bakgrundsdokumentation till SMI:s och RAV:s kunskapsunderlag”Smittsamhet vid behandlad hivinfektion”.

[CR52] The Public Health Agency of Sweden. (2015a). *Contact tracing and notification requirement.* Retrieved from http://www.folkhalsomyndigheten.se/hividag/testning/smittsparning-och-informationsplikt.

[CR53] The Public Health Agency of Sweden. (2015b). *Att leva med HIV i Sverige*—*En studie om livskvalitet hos personer som lever med HIV* [Living with HIV in Sweden—A quality of life study among people living with HIV]. Retrieved from http://www.folkhalsomyndigheten.se/publiceratmaterial/publikationer/Att-leva-med-hiv-i-Sverige–En-studie-om-livskvalitet-hospersoner-som-lever-medhiv.

[CR54] Tng, J. (2016). SFU study explores stigma faced by HIV-positive women. A Q&A with SFU assistant professor Angela Kaida. *The Peak*. Retrieved from http://the-peak.ca/2016/02/sfu-study-explores-stigma-faced-by-hiv-positive-women/.

[CR55] Velten J, Margraf J (2017). Satisfaction guaranteed? How individual, partner, and relationship factors impact sexual satisfaction within partnerships. PLoS ONE.

[CR56] Vincent W, Fang X, Calabrese SK, Heckman TG, Sikkema KJ, Hansen NB (2016). HIV-related shame and health-related quality of life among older, HIV-positive adults. Journal of Behavioral Medicine.

[CR57] Weatherburn P, Keogh P, Reid D, Dodds C, Bourne A, Owuor J, Hammond G, Jessup K (2009). What do you need? 2007–08 findings from a national survey of people with diagnosed HIV.

[CR58] Wilson TE, Jean-Louis G, Schwartz R, Golub ET, Cohen MH, Maki P, Greenblatt R, Massad LS, Robison E, Goparaju L, Lindau S (2010). HIV infection and women’s sexual functioning. Journal of Acquired Immune Deficiency Syndromes.

[CR59] World Health Organization. (2013). *Treatment and prevention.* Retrieved from http://www.who.int/hiv/pub/guidelines/arv2013/download/en/.

[CR60] World Health Organization. (2016). *Defining sexual health.* Retrieved from http://www.who.int/reproductivehealth/topics/sexual_health/sh_definitions/en/.

